# Protective Effects of Li-Fei-Xiao-Yan Prescription on Lipopolysaccharide-Induced Acute Lung Injury via Inhibition of Oxidative Stress and the TLR4/NF-*κ*B Pathway

**DOI:** 10.1155/2017/1791789

**Published:** 2017-03-23

**Authors:** Lie-Qiang Xu, Xiu-Ting Yu, Shu-Hua Gui, Jian-hui Xie, Xiu-Fen Wang, Zu-Qing Su, Yu-Cui Li, Xiao-Ping Lai, Janis Ya-Xian Zhan, You-Liang Xie

**Affiliations:** ^1^School of Chinese Materia Medica, Guangzhou University of Chinese Medicine, Guangzhou, China; ^2^Guangdong Provincial Key Laboratory of New Drug Development and Research of Chinese Medicine, Guangzhou University of Chinese Medicine, Guangzhou 510006, China; ^3^The First Affiliated Hospital of Chinese Medicine, Guangzhou University of Chinese Medicine, Guangzhou, China; ^4^Guangdong Provincial Hospital of Chinese Medicine, Guangzhou, China

## Abstract

Li-Fei-Xiao-Yan prescription (LFXY) has been clinically used in China to treat inflammatory and infectious diseases including inflammatory lung diseases. The present study was aimed at evaluating the potential therapeutic effects and potential mechanisms of LFXY in a murine model of lipopolysaccharide- (LPS-) induced acute lung injury (ALI). In this study, the mice were orally pretreated with LFXY or dexamethasone (positive drug) before the intratracheal instillation of LPS. Our data indicated that pretreatment with LFXY enhanced the survival rate of ALI mice, reversed pulmonary edema and permeability, improved LPS-induced lung histopathology impairment, suppressed the excessive inflammatory responses* via* decreasing the expression of proinflammatory cytokines (TNF-*α*, IL-1*β*, and IL-6) and chemokine (MIP-2) and inhibiting inflammatory cells migration, and repressed oxidative stress through the inhibition of MPO and MDA contents and the upregulation of antioxidants (SOD and GSH) activities. Mechanistically, treatment with LFXY significantly prevented LPS-induced TLR4 expression and NF-*κ*B (p65) phosphorylation. Overall, the present study suggests that LFXY protected mice from acute lung injury induced by LPS* via* inhibition of TLR4/NF-*κ*B p65 activation and upregulation of antioxidative enzymes and it may be a potential preventive and therapeutic agent for ALI in the clinical setting.

## 1. Introduction

Acute lung injury (ALI) and its more severe form, acute respiratory distress syndrome, are defined as severe complications with systemic inflammatory responses in the air spaces and lung parenchyma [1]. The pathogenesis of ALI can be characterized by inflammatory injury of the alveolar-capillary membrane, increase of pulmonary vasculature permeability, neutrophils accumulation, and pulmonary edema [2]. ALI is a life-threatening problem with an associated mortality of 40–80% [3]. Therefore, there is an urgent need to look for a novel therapeutic strategy for the management of ALI.

Lipopolysaccharide (LPS), a principal component of the outer membranes of Gram-negative bacteria, has been widely used to induce ALI in animal models [4]. Toll-like receptor 4 (TLR4), a transmembrane receptor, acts as a critical sensor to recognize LPS and activates the downstream signals. TLR4 triggers the activation of nuclear factor-*κ*B (NF-*κ*B) signaling pathway, which induces the production of proinflammatory cytokines and chemokines, such as tumor necrosis factor-*α* (TNF-*α*), interleukin-6 (IL-6), interleukin-1*β* (IL-1*β*), and macrophage inflammatory protein-2 (MIP-2), and subsequently leads to the recruitment of inflammatory cells in the lung and finally leads to the occurrence of ALI [5–7]. Moreover, LPS-induced accumulation of neutrophils brings about the production of reactive oxygen species (ROS). Generally, the ROS level is regulated by the antioxidants such as superoxide dismutase (SOD), catalase (CAT), and glutathione peroxidase (GSH-Px) [8, 9]. However, once excessive ROS overwhelms endogenous antioxidant defense systems, oxidative stress occurs in the lung tissue. Oxidative stress induces tissue damage and triggers inflammasome activation, resulting in severe inflammation in ALI [10]. Based on the underlying mechanisms of ALI, it is believed that antioxidant and anti-inflammatory agents may serve as effective therapeutic strategies for the treatment of ALI.

Qing-Yan-Li-Ge Tang, a traditional Chinese herbal prescription, is used for the treatment of pharyngitis attributed to its anti-inflammatory effects and has advantages in the treatment of acute pharyngitis, acute tonsillitis, retropharyngeal abscess, and so forth [11]. It was first recorded in the ancient Chinese medical book* Hou Ke Zi Zhen Ji* by Xiangyu Zhu (1860, Qing Dynasty). According to Chinese medicinal theory, Li-Fei-Xiao-Yan (LFXY) is derived from Qing-Yan-Li-Ge Tang and is composed of 10 medicinal herbs (as shown in Table 1). Most of these constituent herbs have been reported to have antioxidant and anti-inflammatory activities, such as* Lonicera japonica* Thunb.,* Scutellaria baicalensis* Georgi,* Gardenia jasminoides* Ellis,* Scrophularia ningpoensis* Hemsl.,* Forsythia suspensa* (Thunb.) Vahl, and* Glycyrrhiza uralensis* Fisch. Among them,* Scutellaria baicalensis* Georgi, one of the main herbs of this formula, has been proven to be effective in treating pulmonary disease [12]. According to Shin et al. [13], heat-processed* Scutellaria* radix could attenuate acute lung injury induced by LPS in mice via NF-*κ*B signaling. Baicalein, one of the main ingredients of* Scutellaria baicalensis*, was reported to protect against LPS-induced ALI in rats by remarkably reducing IL-1*β*, TNF-*α*, and IL-6 levels [14].

Based on these observations, we hypothesize that LFXY might have a potential in the treatment of ALI due to its antioxidant and anti-inflammatory activities. To experimentally test this hypothesis, we investigate the possible protective effects of LFXY against LPS-induced ALI in mice and delineate its potential mechanisms in the present work.

## 2. Methods

### 2.1. Materials and Chemicals

The medicinal herbs in LFXY (shown in Table 1) were purchased from Guangxi Yifang Chinese Herbal Medicine Department (Guangxi, China) and identified by Professor Lai Xiaoping (pharmacognosist of the School of Chinese Materia Medica, Guangzhou University of Chinese Medicine). Voucher specimens were deposited in the Herbarium of Guangzhou University of Chinese Medicine with voucher specimen numbers assigned as DZCM 2015-045 (*Scutellaria baicalensis *Georgi), DZCM 2015-012 (*Lonicera japonica *Thunb.), DZCM 2015-013 (*Forsythia suspensa *(Thunb.) Vahl), DZCM 2015-020 (*Gardenia jasminoides *Ellis), DZCM 2015-067 (*Schizonepeta tenuifolia *Briq.), DZCM 2015-039 (*Scrophularia Ningpoensis *Hemsl.), DZCM 2015-007 (*Saposhnikovia divaricata *(Turcz.) Schischk.), DZCM 2015-011 (*Mentha haplocalyx *Briq.), DZCM 2015-035 (*Glycyrrhiza uralensis *Fisch.), and DZCM 2015-068 (*Belamcanda chinensis *(L.) DC.). Lipopolysaccharide (LPS) and dexamethasone were purchased from Sigma Co. Ltd. (St. Louis, USA) and Huanan Pharmaceutical Group Co., Ltd. (Guangdong, China), respectively. Kits used for determination of SOD, MDA, MPO, GSH, and BCA protein assays were purchased from the Nanjing Jiancheng Bioengineering Institute (Nanjing, Jiangsu, China). Mouse TNF-*α*, IL-1*β*, IL-6, and MIP-2 enzyme-linked immunosorbent assay kits were obtained from eBioscience (CA, USA). Antibodies against TLR4, NF-*κ*B p65, and *β*-actin and secondary antibodies were purchased from Santa Cruz Biotechnology (Santa Cruz, CA, USA). All other chemicals were of analytic grade.

### 2.2. Preparation and Chemical Analysis of LFXY

LFXY was composed of 10 kinds of herbal medicines (Table 1). Briefly, the root of* Scutellaria baicalensis* Georgi was extracted twice with 80% aqueous ethanol for 2 h, evaporated to dryness under reduced pressure, and then dissolved in distilled water and filtered. Essential oils of* Lonicera japonica *Thunb.,* Schizonepeta tenuifolia* Briq.,* Mentha haplocalyx *Briq., and* Saposhnikovia divaricata* (Turcz.) Schischk. were extracted with water vapor, respectively, and then the residues as well as another 5 herbal materials were mixed and refluxed for 2 h with 60% ethanol. After reflux extraction, the ethanol extract solution was filtered and concentrated and then we mixed it up with volatile oils and the water solution of* Scutellaria baicalensis* Georgi. Finally, the mixture was diluted with water to the volume of 1000 mL to obtain a final concentration of 194.2 mg/mL.

In this study, HPLC analysis of LFXY was carried out on a Shimadzu LC-20A HPLC system consisting of an SPD-M20A PDA detector, an LC-20AD pump, an SIL-20AC automatic sampler, and CTO-20A thermostatic column compartment (Shimadzu, Kyoto, Japan). Chromatographic separation was performed at 35°C with a flow rate of 1 mL/min on a Diamonsil-C_18_ (250 mm × 4.6 mm, 5 *μ*m). The mobile phase consisted of acetonitrile (as solvent A) and 0.1% phosphoric acid (as solvent B). The elution program was optimized and conducted as follows: 0–30 min, 5–19% A; 30–40 min, 19% A; 40–60 min, 19–40% A; 60–90 min, 40–45% A; 90–115 min, 45–50% A; 115–125 min, 50–55% A. A preequilibration period of 20 min was used between individual runs. The signal was detected at a wavelength of 254 nm.

### 2.3. Animals

Since KM mice have been widely used in LPS-induced ALI model [15–18], this species was therefore employed in the present work for the in vivo assay. Male Kunming (KM) mice (18–22 g) were obtained from the Medical Laboratory Animal Center of Guangdong Province, China. All the animals were maintained under environmentally controlled conditions of 23 ± 2°C, with relative humidity of 55 ± 10%, under a 12-hour light/dark cycle with free access to standard laboratory diet and water. The animal experiments were approved by the Animal Ethics Committee of Guangzhou University of Chinese Medicine (approval number Scxk 2015-0068) and the animal received humane care in accordance with the* Guide for the Care and Use of Laboratory Animals*, published by the US National Institutes of Health (NIH Publication, revised in 1996).

### 2.4. Experimental Design

Firstly, the mortality rate of ALI mice was assessed. Mice were assigned randomly to the following 5 groups (10 mice per group): sham group, LPS group, and LPS + LFXY groups (25, 50, and 100 mg/kg body weight). Mice in LPS + LFXY group received intragastric administration of LFXY at the corresponding dose once daily for 5 consecutive days, while sham group and LPS group were orally given the same volume of saline. One hour later after the last administration, all mice were anaesthetized with 3% chloral hydrate via intraperitoneal injection. Subsequently, LPS (35 mg/kg, 20 *μ*L/10 g body weight) was intratracheally administered into mice in LPS group and LPS + LFXY groups to induce ALI, while mice in the sham group received the same volume of phosphate buffered saline (PBS, 20 *μ*L/10 g body weight). Then, the mortality of mice was calculated every 6 h up to 144 h.

Secondly, animals were divided into 6 groups randomly (20 mice per group): sham group, LPS group, LPS + LFXY groups (25, 50, and 100 mg/kg), and LPS + DEX group (5 mg/kg dexamethasone, positive drug). Before ALI challenge, treatment groups were orally pretreated with LFXY or dexamethasone (DEX). Sham and LPS mice were given the same volume of saline. After 1 h, mice were slightly anaesthetized with 3% chloral hydrate via intraperitoneal injection. ALI was induced by intratracheal administration of LPS (5 mg/kg, 20 *μ*L/10 g body weight). During this period, sham mice were given the same volume of PBS (20 *μ*L/10 g body weight).

For the specimen collection, 24 h after LPS instillation, mice from all groups were sacrificed humanely by lethal sodium pentobarbital injection. In each group, the left lung (*n* = 10) was removed for wet-to-dry (W/D) weight ratio analysis, and the right lung was removed for western blot and histopathological assays. In addition, the left lungs from the rest of the mice were collected for the analysis of MPO, SOD, GSH, and MDA levels, and bronchoalveolar lavage fluid (BALF) was obtained with PBS from the right lung for cytokines and leukocyte content assays.

### 2.5. Body Weight Measurement

In mortality rate study and pharmacodynamic evaluation, all mice's final body weights were recorded before they were sacrificed humanely or died after LPS challenge. All data was calculated to show the effect of LFXY on body weight.

### 2.6. Lung W/D Weight Ratio

The mice were humanely sacrificed by lethal sodium pentobarbital injection 24 h after LPS stimulation. Briefly, the left lung tissues were collected and immediately weighed to record the “wet” weight and then dried in an oven (for 48 h at 80°C) to stabilize “dry” weight. Then, the W/D weight ratio was calculated by the “wet” weight to the stable “dry” weight to assess the severity of pulmonary edema [19].

### 2.7. Histological Study

For histological examination, the tissue were fixed in 10% buffered formalin, embedded in paraffin, and cut into 5 *μ*m thickness. Hematoxylin and eosin staining was subsequently carried out using standard histological techniques. Evaluation of lung injury was then performed using these histological preparations under a light microscope. The severity of lung injury was scored using a published scoring system in a blinded manner [20]. Four categories of lung pathological changes, that is, infiltration of inflammatory cells, edema, hemorrhage, and alveolar wall thickening, were used to evaluate the severity of lung injury and each item was scored on a scale of 0 (normal) to 4 (severe). The total lung injury score was expressed as the sum of the four criteria.

### 2.8. Measurement of BALF Protein Contents and Cell Counts

The BALF was centrifuged (4°C, 800 ×g, 10 min) and the sediment cells were resuspended in PBS for the total cell counts using a hemacytometer, and cytospins were stained by a Wright-Giemsa staining kit (Jiancheng Company, Nanjing, China) for the differential cell counts [21]. The supernatants were stored at −20°C until used for protein assay.

### 2.9. Determination of MPO Activity

MPO is an index of neutrophil accumulation in the tissue and correlates with the severity. MPO activity in lung tissue was determined using a MPO activity kit based on the manufacturer's instruction. The enzymatic activity was determined by using a 96-well plate reader at 460 nm. The activity was expressed as units per gram of protein.

### 2.10. Measurement of MDA, SOD, and GSH Levels in Lung

Lung tissues were homogenized in PBS (pH 7.4) and then centrifuged (12000 rpm, 4°C, 10 min), and the supernatant was prepared for the GSH, SOD, and MDA assays using commercially available assay kits according to the manufacturer's instructions, respectively.

### 2.11. Determination of TNF-*α*, IL-6, IL-1*β* , and MIP-2 in BALF

Based on the manufacturer's directions, the concentrations of cytokines and chemokines including TNF-*α*, IL-6, IL-1*β*, and MIP-2 in BALF were evaluated by mouse ELISA kits. The results were equated against standard curves and expressed as pg/mL of each cytokine.

### 2.12. Western Blot Analysis

24 h after LPS challenge, lung tissues were collected and immediately frozen in liquid nitrogen for storage until homogenization. The protein was extracted using T-PER Tissue Protein Extraction Reagent Kit. And concentrations were measured using a BCA protein assay kit according to the manufacturer's protocol. And equivalent amounts of protein were subjected to 10% sodium dodecyl sulphate polyacrylamide gel (SDS-PAGE) and electrophoretically transferred to polyvinylidene difluoride (PVDF) membranes. The resulting membranes were blocked with Tris-buffered saline containing 0.05% Tween-20 (TBS-T) and 5% skimmed milk for 2 h and then washed with TBS-T. The specific primary antibodies anti-NF-*κ*B p65 and anti-TLR4 diluted (1 : 1000, v/v) in TBS-T were incubated with the membrane at 4°C overnight and then washed with TBS-T and incubated with the peroxidase-conjugated secondary antibody at room temperature for 1 h. The immune active proteins were detected by using an enhanced-chemiluminescence (ECL) western blotting detection kit. And the relative protein levels were normalized to *β*-actin protein as the internal standard.

### 2.13. Statistical Analysis

All data were calculated as mean ± SEM. Comparisons of mean values in different groups were performed by one-way analysis of variance (ANOVA) followed by Student-Newman-Keuls test. Statistical analyses were finished by statistical analysis software (SPSS 17.0). Results with a *P* < 0.05 were regarded as statistically significant.

## 3. Results

### 3.1. HPLC Analysis of LFXY

**Figure 1:**
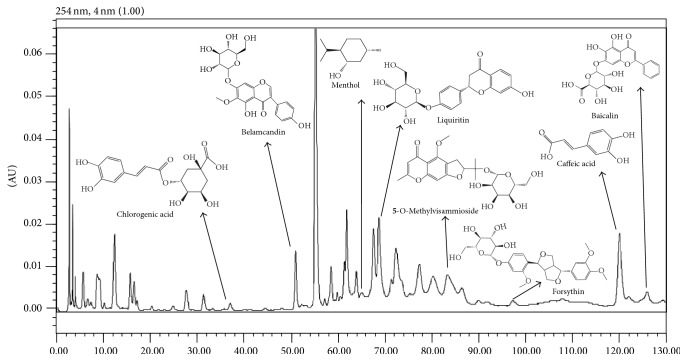
The HPLC analysis of the main components in LFXY. Identified compounds were compared with the retention times and UV spectra of the reference standards.

The proposed HPLC analytical method was applied to detect the main components in LFXY. As depicted in Figure 1, 8 compounds (namely, chlorogenic acid, caffeic acid, 5-O-methylvisammioside, belamcandin, baicalin, forsythin, menthol, and liquiritin) in LFXY were identified by comparing the retention times and UV spectra of the reference standards.

### 3.2. Effects of LFXY on Body Weight

LPS challenge leads to the reduction of activity and food intake. As shown in Table 2, in pharmacodynamic evaluation, there were no significant differences among each group after 5 consecutive days of LFXY treatment. However, in mortality rate study, after 5 consecutive days of LFXY treatment, LPS was intratracheally administered into mice. We found that the body weight obviously decreased after that; this deteriorated alternation was observed to be mitigated in mice of LFXY groups.

### 3.3. Effects of LFXY on LPS-Induced Mortality in ALI Mice

**Figure 2:**
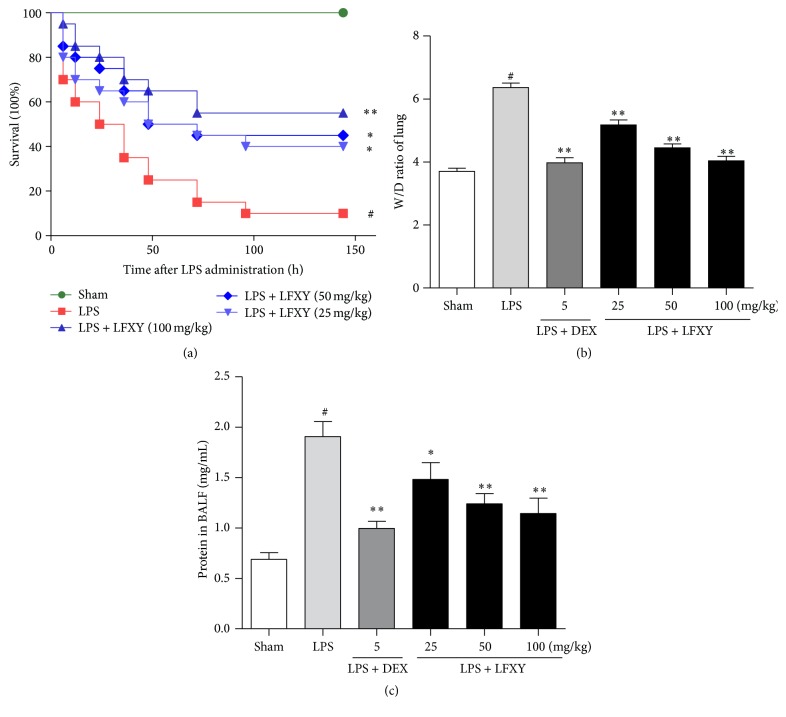
Effects of LFXY on LPS-induced mortality in ALI mice (a), the lung wet/dry ratio (b), and protein contents in BALF (c). Data represented the mean ± SEM (*n* = 10). ^#^*P* < 0.01 versus sham group; ^*∗*^*P* < 0.05 and ^*∗∗*^*P* < 0.01 versus LPS group.

As shown in Figure 2(a), the accumulative mortality during 6 days in LPS group was significantly increased (90%, *P* < 0.01* versus* sham group) compared with the sham group (the mortality of 0%). However, the accumulative mortality was significantly reduced after pretreatment with LFXY or dexamethasone (DEX). The mortalities of mice in LFXY groups (25, 50, and 100 mg/kg) were 60% (*P* < 0.05), 55% (*P* < 0.05), and 45% (*P* < 0.01), respectively, which were evidently lower as compared with those in LPS group (90%). It is suggested that pretreatment with LFXY could significantly protect the mice from LPS-induced death in a dose-dependent manner.

### 3.4. Effects of LFXY on ALI-Induced Lung W/D Ratio and BALF Protein Contents

Pulmonary edema is a typical characteristic of ALI. In the present work, the lung W/D ratio and BALF protein contents were evaluated to quantify the magnitude of pulmonary edema and pulmonary vascular permeability. As illustrated in Figure 2(b), LPS challenge induced a remarkable increase in the lung W/D ratio compared with the sham group (*P* < 0.01), whereas this increase was significantly attenuated by LFXY (25, 50, and 100 mg/kg, *P* < 0.01) and DEX pretreatments (5 mg/kg, *P* < 0.01), thus suggesting the relief of pulmonary edema. As compared with DEX (5 mg/kg), LFXY pretreatment (100 mg/kg) achieved similar results in relieving LPS-induced pulmonary edema. Additionally, LPS treatment significantly increased the protein concentration in BALF (*P* < 0.01), as illustrated in Figure 2(c). However, pretreatment with LFXY (25 mg/kg, *P* < 0.05; 50 and 100 mg/kg, *P* < 0.01) obviously decreased the total protein concentration in BALF when compared with the LPS group, although the ameliorative effect was relatively weaker to the positive control. These results indicate that LFXY could significantly inhibit lung edema and mitigate the pulmonary vascular permeability enhancement in mice with ALI.

### 3.5. Effects of LFXY on LPS-Induced Lung Histopathological Changes in ALI Mice

**Figure 3:**
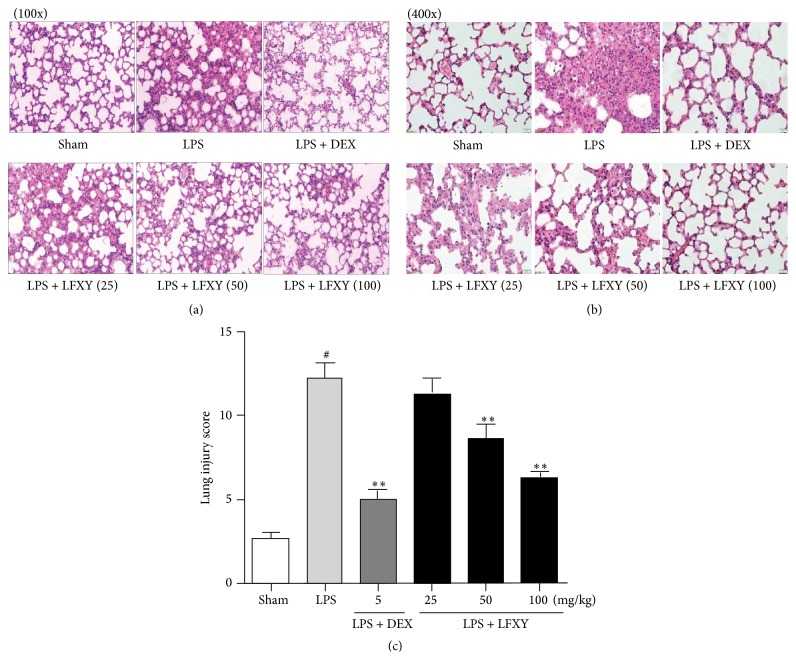
Effects of LFXY on LPS-induced lung histopathological changes ((a) magnification 100x, (b) magnification 400x) and lung injury score (c) in ALI mice. Values represented the mean ± SEM. ^#^*P* < 0.01 versus sham group; ^*∗*^*P* < 0.05 and ^*∗∗*^*P* < 0.01 versus LPS group.

As shown in Figures 3(a) and 3(b), lung sections from the sham group showed normal structure without abnormal histopathological changes. However, under the challenge of LPS, the pulmonary function of mice was obviously impaired, with various pathological changes including infiltration of inflammatory cells, capillary congestion, hemorrhaging, and marked thickening of the alveolar wall. However, these changes were obviously abated by pretreatment with LFXY (25, 50, and 100 mg/kg) and DEX (5 mg/kg), when compared to the LPS model group. Moreover, a similar inhibitory effect was found in semiquantitative analysis by lung injury score, which presented the evaluation of severity of ALI (Figure 3(c)). Results demonstrated that pretreatment with LFXY could significantly attenuate the severity of lung injuries of ALI mice induced by LPS and improved the impairments of lung tissues.

### 3.6. Effects of LFXY on Inflammatory Cell Counts in BALF

**Figure 4:**
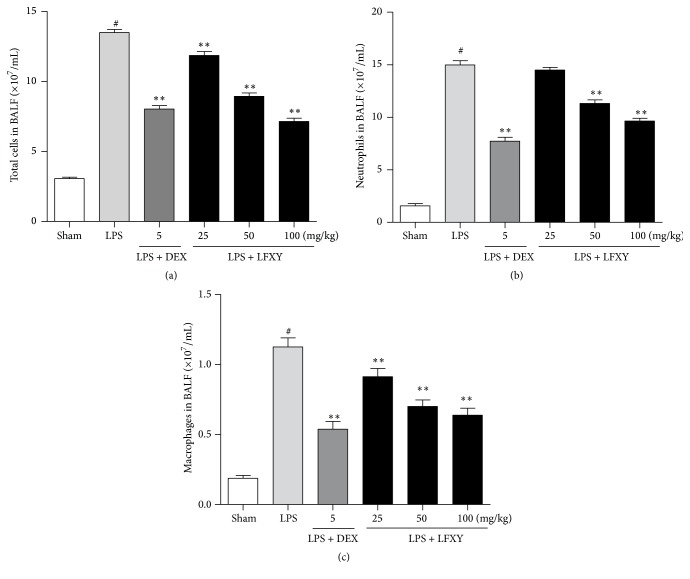
Effects of LFXY on the inflammatory cell count in BALF in BALF. (a) Total cells, (b) neutrophils, and (c) macrophages. Values represented the mean ± SEM. ^#^*P* < 0.01 versus sham group; ^*∗*^*P* < 0.05 and ^*∗∗*^*P* < 0.01 versus LPS group.

The number of inflammatory cells, such as neutrophils and macrophages, in BALF was counted 24 h after treatment with LPS. As shown in Figures 4(a), 4(b), and 4(c), LPS challenge significantly increased the number of total cells, neutrophils, and macrophages as compared with the sham group (*P* < 0.01). However, pretreatment with LFXY (25, 50, and 100 mg/kg, *P* < 0.01) and DEX (5 mg/kg, *P* < 0.01) significantly decreased the number of total cells and macrophages in BALF. And intragastric administration of LFXY (100 mg/kg) was shown to exert a similar effect to DEX (5 mg/kg). In addition, LFXY at doses of 50 and 100 mg/kg resulted in a marked decrease in the level of neutrophils (*P* < 0.01). These results indicate that LFXY pretreatment could protect mice from LPS-induced inflammation in ALI.

### 3.7. Effect of LFXY on MPO, MDA, SOD, and GSH Levels in Lung Tissues of Mice with ALI

**Figure 5:**
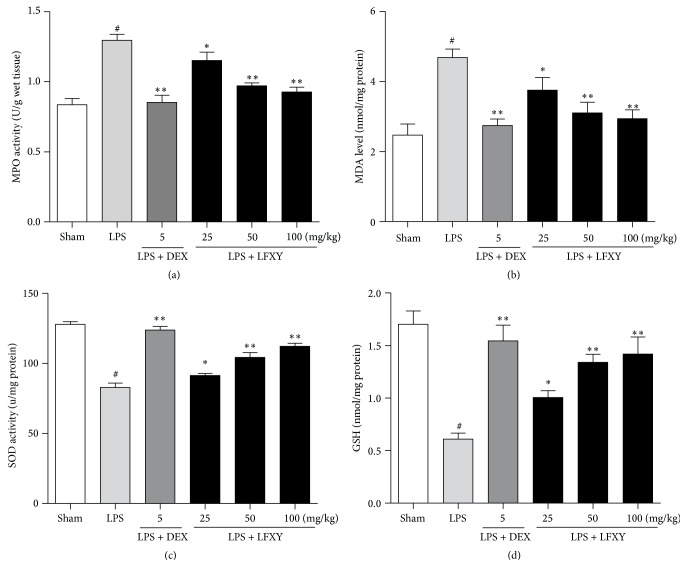
Effects of LFXY on MPO, MDA, SOD, and GSH levels in lung tissues. (a) MPO activity (units per gram of wet tissue). (b) MDA level (nmol/mg protein). (c) SOD activity (units/mg protein). (d) GSH activity (nmol/mg protein). Values represented the mean ± SEM. ^#^*P* < 0.01 versus sham group; ^*∗*^*P* < 0.05 and ^*∗∗*^*P* < 0.01 versus LPS group.

As shown in Figures 5(a) and 5(b), LPS stimulation led to significant increases in lung MPO activity and MDA level compared with the sham group. However, these increases were markedly reversed when mice were pretreated with LFXY (25 mg/kg, *P* < 0.05; 50 and 100 mg/kg, *P* < 0.01). A similar inhibitory effect was found in DEX (5 mg/kg, *P* < 0.01). In addition, SOD and GSH activities were significantly reduced after LPS challenge compared to those in the sham group, while pretreatment with LFXY (25 mg/kg, *P* < 0.05; 50 and 100 mg/kg, *P* < 0.01) and DEX (5 mg/kg, *P* < 0.01) substantially enhanced the SOD and GSH activities compared to the LPS group (Figures 5(c) and 5(d)), and the effect in high dose of LFXY (100 mg/kg) was comparable to that of 5 mg/kg DEX.

### 3.8. Effects of LFXY on the Production of TNF-*α*, IL-6, IL-1*β*, and MIP-2 in BALF

**Figure 6:**
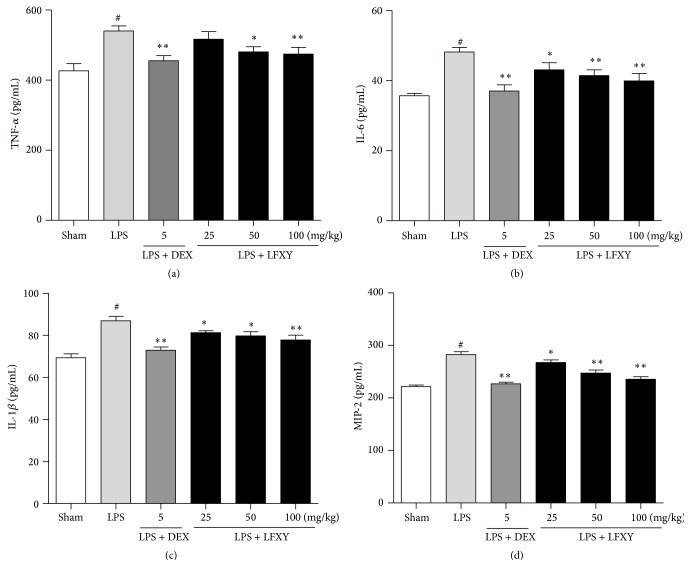
Effects of LFXY on the production of TNF-*α*, IL-6, IL-1*β*, and MIP-2 in BALF. BALF was collected 24 h after LPS challenge and the concentrations of TNF-*α* (a), IL-6 (b), IL-1*β* (c), and MIP-2 (d) in BALF were determined. Data presented were mean ± SEM. ^#^*P* < 0.01 versus sham group; ^*∗*^*P* < 0.05 and ^*∗∗*^*P* < 0.01 versus LPS group.

The effects of LFXY on TNF-*α*, IL-6, IL-1*β*, and MIP-2 concentrations in BALF were analyzed by ELISA 6 h after LPS stimulation. As shown in Figure 6, the concentrations of TNF-*α*, IL-6, IL-1*β*, and MIP-2 in BALF were obviously elevated in the LPS group compared with those in the sham group. However, pretreatment with LFXY reversed the levels of TNF-*α*, IL-6, IL-1*β*, and MIP-2 (*P* < 0.05 or *P* < 0.01). DEX was also shown to exert a significant inhibitory effect on the production of these cytokines and chemokines in BALF (*P* < 0.01).

### 3.9. Effect of LFXY on TLR4 and NF-*κ*B Expressions in Acute Lung Injury Mice Induced by LPS

**Figure 7:**
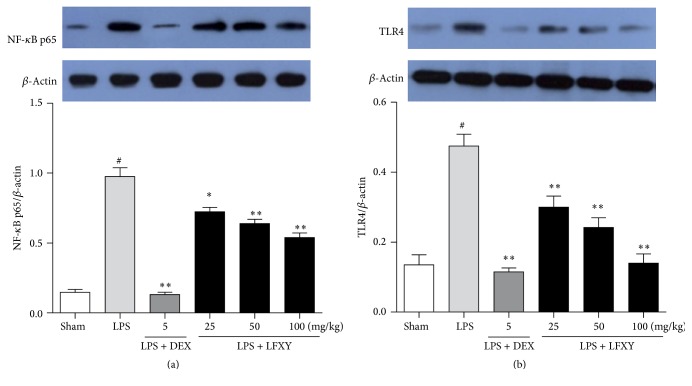
Effects of LFXY on the expression of TLR4 and NF-*κ*B p65 in the lung tissue by western blot assay. The expression of NF-*κ*B p65 (a) and TLR4 (b). Data presented were mean ± SEM. ^#^*P* < 0.01 versus sham group; ^*∗*^*P* < 0.05 and ^*∗∗*^*P* < 0.01 versus LPS group.

TLR4/NF-*κ*B signaling pathway induces the production of most proinflammatory cytokines including TNF-*α*, IL-1*β*, and IL-6, thus playing an important role in the pathogenesis of ALI. Western blot analysis was performed to determine the phosphorylation of NF-*κ*B p65 and the expression of TLR4. As shown in Figure 7, LPS administration significantly increased the phosphorylation of NF-*κ*B p65 in the lung, as compared with the sham group. However, pretreatment with LFXY (25, 50, and 100 mg/kg) inhibited the phosphorylation of NF-*κ*B p65 in a dose-dependent manner. In addition, the expression of TLR4 was also observed to be significantly increased in the LPS-treated mice, while the tendency was dramatically attenuated by pretreatment with LFXY (25, 50, and 100 mg/kg). These results show that LFXY pretreatment could block the TLR4/NF-*κ*B signaling pathway in a mouse model of LPS-induced ALI.

## 4. Discussion

The development of ALI can be attributed to infection of LPS [1]. In the mouse model of LPS-stimulated ALI, the symptoms are nearly consistent with the pathological characteristics of clinical ALI in human, including the disruption of endothelial and epithelial integrity, lung edema, and the release of inflammatory mediators [3]. Mounting study highlights that inflammatory response and oxidative stress are important factors in the pathogenesis of ALI [7]. Therefore, inhibition of LPS-induced inflammatory responses and oxidative stress is critical for the treatment of ALI. Interestingly, most herbal medicines in the LFXY formula show antioxidative and anti-inflammatory activities. These lines of evidence indicate that LFXY may be a promising potential therapeutic natural material to protect the lung against LPS-induced ALI. Therefore, this study investigated the protective effects of LFXY in a LPS-induced ALI mouse model.

LPS challenge leads to the influx of serous fluids into lung tissues, which leads to alveolar-capillary edema, one of the major characteristics of ALI [22, 23]. The W/D ratio is a commonly used indicator for pulmonary edema [20]. In the present study, we examined significant inhibition of the W/D ratio in LFXY-treated groups. Besides, the lung permeability, evaluated by the total protein concentration in BALF, was relieved by pretreatment with LFXY. LPS challenge also directly stimulated the infiltrations of inflammatory cells, especially the neutrophils [24, 25]. MPO, an important index of neutrophil migration into the lung, reflects the presence of neutrophils in lung tissues [4]. In addition, macrophages play a vital role in the recruitment of neutrophils [26]. Consistent with a previous study, we found that MPO activity and the number of neutrophils and macrophages were increased dramatically in the LPS group. However, these changes were significantly mitigated by pretreatment with LFXY. Moreover, the histological analysis of the lungs further revealed that the infiltrations of neutrophils and the lung tissue injuries were ameliorated by LFXY treatment. These findings suggest that LFXY protects mice from lung edema, increase in pulmonary vascular permeability, and acute inflammatory responses in the lung.

Recent experimental studies suggest that intratracheal administration of LPS promotes the production of inflammatory cytokines (such as TNF-*α*, IL-1*β*, and IL-6) and chemokine (such as MIP-2) [27]. These excessive inflammatory cytokines and chemokine are important characteristics of ALI [28]. They not only amplify the inflammatory responses and result in inflammatory injury, but also recruit neutrophils into the lung [29–31]. In the development of inflammation, TNF-*α* can directly damage the vascular endothelial cell and stimulate the mononuclear macrophages to secrete inflammatory factors like IL-6, and thus it is regarded as the earliest and most representative endogenous mediator [32–35]. IL-6 is one of the most common inflammatory cytokines and represents a significant predictor of the severity of ARDA [27, 36]. IL-1*β*, one of the key factors in the pathogenesis of ALI, can lead to the loss of surface active substance, increases alveolar permeability, and finally causes protein exudation [37]. Besides, MIP-2 impels the leukocyte migration to the lungs and then induces the pulmonary inflammation [38]. Thus, inhibition of these inflammatory mediators can help to alleviate the damage caused by ALI. In the present study, we found that the increased levels of these proinflammatory cytokines and chemokines in BALF of the LPS group were attenuated by pretreatment with LFXY. It is probable that the protective effect of LFXY on LPS-induced ALI is* via* inhibition of these severe inflammatory responses.

The transcription factor NF-*κ*B plays a positive role in the regulation of inflammatory and immune responses in LPS-induced ALI [39]. In general, NF-*κ*B dimmers are retained in the cytoplasm in an inactive form, complexed with an inhibitory protein from the I*κ*B family [40]. TLR4 acts as a vital upstream sensor for LPS from pathogens and microorganisms [20], resulting in triggering the activation of NF-*κ*B. Upon TLR4-mediated activation, NF-*κ*B is released from the I*κ*B, incurring p65 translocation into the nucleus to regulate and promote the transcription of inflammatory mediator genes, leading to the release of various cytokines such as TNF-*α*, IL-1*β*, and IL-6 [19, 26, 41]. To further elucidate the possible molecular mechanisms of LFXY in LPS-induced ALI, we investigated whether the anti-inflammatory activity of LFXY was associated with the TLR4/NF-*κ*B pathway. In the present work, it was observed that LFXY pretreatment significantly repressed the expression of TLR4 and the phosphorylation level of NF-*κ*B p65, which corresponded with the variation of proinflammatory cytokines in lung tissues of ALI mice. Therefore, it is suggested that reduction of proinflammatory cytokines and attenuation of pulmonary inflammatory responses by LFXY are probably due to the repression of TLR4 and inhibition of NF-*κ*B signaling pathway.

Oxidative stress in lung tissue has been also considered to be important contributors to the pathogenesis of ALI [42]. ROS can cause serious damage in cellular structure and function via DNA damage, lipid peroxidation, and activation of transcription factors such as NF-*κ*B [43]. MDA is a good indicator of lipid peroxidation and, to some extent, illustrates the level of oxidative stress [8, 44]. Generally, excessive ROS can be removed through enzymatic components such as SOD and nonenzymatic components such as GSH [44]. SOD, an endogenous free radical-scavenging agent, catalyzes the conversion of superoxide radical anions into peroxides [45]. GSH functions as an important intracellular antioxidant and redox regulator converting H_2_O_2_ into nontoxic products such as H_2_O [46]. In this study, we found that pretreatment with LFXY enhanced the SOD and GSH activities and decreased the contents of MDA in mouse lung parenchyma compared with the LPS model group. These data show that the remarkable inhibition of LFXY on ALI induced by LPS might be closely related to its antioxidant activity.

Taking the HPLC results into consideration, we hypothesized that the protective effect of LFXY on LPS-induced ALI could be due to the bioactivities of its medicinal herbs and chemical compounds.* Scutellaria baicalensis* Georgi has been proven to treat pulmonary disease via reducing the levels of proinflammatory cytokines and suppressing NF-*κ*B signaling pathway [13].* Lonicera japonica* has been used to clear away the heat evil and to treat exopathogenic wind heat, epidemic febrile diseases, and several infectious diseases, especially pneumonia in clinic [47], while its chemical marker chlorogenic acid was previously reported to exert both antioxidative and free radical-scavenging activities in vitro [48]. Iridoid glycosides (e.g., geniposide, gardenoside) were reported to be major ingredients responsible for the biological activities of* Gardenia* fruit and its preparations [49, 50]. Xie et al. have also reported that geniposide could exert anti-inflammatory effects through suppressing the expression and release of the inflammatory factors including TNF-*α*, IL-6, NO, and PGE_2_ [25]. Hence, medicinal herbs and chemical compounds in LFXY, which possess potential antioxidative and anti-inflammatory activities, are crucial for the protective effects of LFXY against LPS-induced ALI.

Our study showed that LFXY had significantly protective effect on LPS-induced ALI, which confirmed the prominent anti-inflammatory properties of LFXY. This anti-inflammatory effect of LFXY was comparable to DEX, a widely clinical-used anti-inflammatory agent. According to our literature review, high doses and prolonged use of DEX induce side effects, such as reduction in tetanic stimuli-induced force, muscular atrophy, and neurobehavioral problems [51, 52]. Hence, there is an urgent need to develop novel effective medications for prevention and therapies. Nowadays, traditional Chinese medicine has gradually gained popularity among the world for its high efficiency in treating chronic and complex diseases with the advantage of multitarget therapy. LFXY is derived from Qing-Yan-Li-Ge Tang, a famous formula used to treat respiratory inflammation for its high efficacy and low side effect. Hence, the observations in this study suggest LFXY as an alternative agent for prevention and therapeutic treatment of ALI among DEX.

## 5. Conclusion

**Figure 8:**
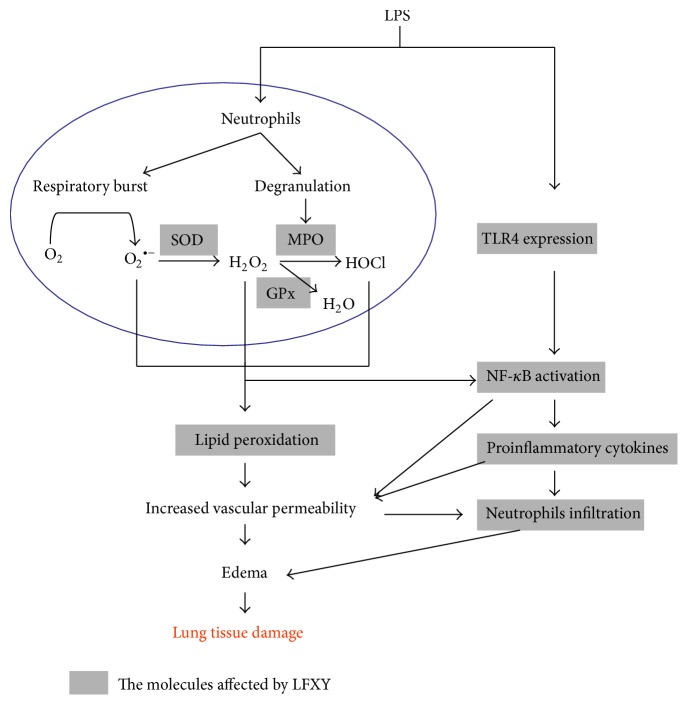
Schemes of the mechanism for the protective effect of LFXY on LPS-induced ALI. The shaded parts indicate the molecules affected by LFXY.

This study demonstrates that LFXY could attenuate LPS-induced lung injury. The protective effect of LFXY is shown to result from inhibiting inflammatory response and oxidative stress and is also associated with the downregulation of TLR4/NF-*κ*B signaling pathway (Figure 8). Thus, our findings suggested that LFXY might act as a potential therapeutic agent for treatment of ALI.

## Supplementary Material

In order to confirm the acute lung histopathological changes induced by LPS and protective effect of LFXY, three photos (H&E stain) for each group with different magnificantion (×100, ×200, ×400) are provided. As shown in supplementary material, we found that LPS administration significantly lead to a acute lung injury and LFXY treatment could improved the impairments of lung tissues.

## Figures and Tables

**Table 1:** Components of Li-Fei-Xiao-Yan prescription (LFXY).

Pictures	Components	Weight (g)	Chemical marker
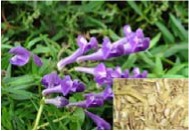	*Scutellaria baicalensis *Georgi (radix)	24	Baicalin
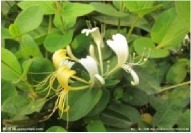	*Lonicera japonica *Thunb. (flower)	24	Chlorogenic acid
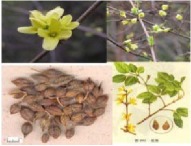	*Forsythia suspensa *(Thunb.) Vahl (fruit)	24	Forsythin
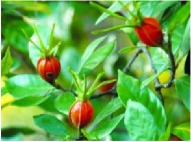	*Gardenia jasminoides *Ellis (fruit)	24	Gardenoside
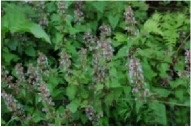	*Schizonepeta tenuifolia *Briq. (aerial part)	24	Menthone
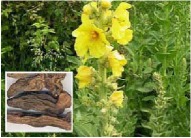	*Scrophularia ningpoensis *Hemsl. (radix)	24	Caffeic acid
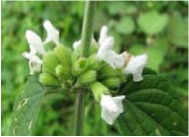	*Saposhnikovia divaricata *(Turcz.) Schischk. (radix)	14.3	5-O-Methylvisammioside
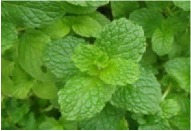	*Mentha haplocalyx *Briq. (aerial part)	14.3	Menthol
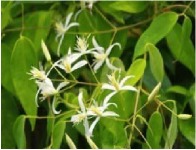	*Glycyrrhiza uralensis *Fisch. (rhizome and radix)	14.3	Liquiritin
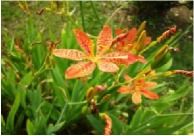	*Belamcanda chinensis *(L.) DC. (rhizome)	7.3	Belamcandin

**Table 2:** Effect of LFXY on body weight.

Group	Mortality rate study (g)	Pharmacodynamic evaluation (g)
Sham	24.8 ± 0.51	23.1 ± 0.68
LPS	20.0 ± 0.67^#^	22.3 ± 0.57
LPS + DEX		22.8 ± 0.39
LPS + LFXY	20.7 ± 0.61	22.3 ± 0.89
LPS + LFXY	22.1 ± 0.60^*∗*^	23.1 ± 0.87
LPS + LFXY	24.3 ± 0.66^*∗∗*^	22.3 ± 0.32

Data are presented as mean ± SEM. ^#^*P* < 0.01 versus sham group. ^*∗*^*P* < 0.05 and ^*∗∗*^*P* < 0.01 versus LPS group.
